# ﻿*Callicarpayongshunensis* (Lamiaceae): A new species from Hunan, China

**DOI:** 10.3897/phytokeys.241.119343

**Published:** 2024-04-22

**Authors:** Wen-Bin Xu, Xiao-Dong Li, Shu-Hui Wang, Ang Liu, Yan-Ling Liu

**Affiliations:** 1 Wuhan Botanical Garden, Chinese Academy of Sciences, Wuhan 430074, Hubei, China Wuhan Botanical Garden, Chinese Academy of Sciences Wuhan China; 2 Central South University of Forestry and Technology, Changsha 410004, Hunan, China Central South University of Forestry and Technology Changsha China

**Keywords:** *Callicarpa*, China, Hunan, Lamiaceae, morphology, new species

## Abstract

This study provides detailed description of a newly-discovered *Callicarpayongshunensis* Wen B. Xu, Xiao D. Li & Yan Ling Liu (Lamiaceae) species from Hunan, China. The species shares similarities in the inflorescence, glandular colour and leaf shape features with *C.luteopunctata* H. T. Chang and *C.giraldii* Hesse ex Rehd., while its white fruits are similar to those of *C.longifolia* Lamk. However, its procumbent, evergreen shrub and white fruits are distinctly different from those of *C.luteopunctata* and *C.giraldii*, while its procumbent, scarless nodes and stellate pubescence free fruits distinguishes it from *C.longifolia*. Images, distribution, morphological features, molecular phylogenetic classification and conservation assessment of this new *Callicarpa* species are explored.

## ﻿Introduction

The genus *Callicarpa* L. was established by Linnaeus (1753), but the family to which it belongs was not defined at that time. Brown (1810) assigned the genus in the family Verbenaceae, which, for decades, was widely recognised by most taxonomists (Endlicher 1836; Schauer 1847; Briquet 1895; Moldenke 1936; Fang 1982; Chen and Michael 1994). However, significant adjustment and delimitation of Lamiaceae and Verbenaceae have been proposed in the past three decades, based on morphological features, especially inflorescence characteristics and molecular systematics (Cantino et al. 1992; Wagstaff and Olmstead 1997; Olmstead et al. 2001; Harley et al. 2004; Ma et al. 2015; Zhao et al. 2021). Consequently, the genus *Callicarpa* was re-assigned into the Lamiaceae family along with the subfamily Viticoideae and some groups that originally belonged to the family Verbenaceae. Recent molecular phylogenetic studies have further indicated that the genus *Callicarpa* belongs to the Lamiaceae family, suggesting that it is sister to the endemic Australian subfamily Prostantheroideae, with both groups being early diverging lineages in the family (Mint Evolutionary Genomics Consortium 2018; Zhao et al. 2021). Species delimitation within the genus is typically based on traits, such as life form, number of inflorescence branches, glandular colour, filament length, mode of anther opening, fruit colour and size, as well as presence or absence of stellate hairs (Fang 1982; Chen and Michael 1994).

The genus *Callicarpa* harbours approximately 140 species and it is distributed in the tropical to temperate Asia, America, Australia, Pacific Islands and Madagascar (Chang 1951; Fang 1982; Chen and Michael 1994; Bramley 2011, 2013; Bramley et al. 2019). A total of 48 species in the genus *Callicarpa* were catalogued in the *Flora* of *China* (Chen and Michael 1994). Later, four new species, including *C.hainanensis* Z. H. Ma & D. X. Zhang (Ma and Zhang 2012), *C.pararubella* Qiang Wang (Wang 2019), *C.stoloniformis* X. X. Su, Z. H. Ma & B. Chen (Ma et al. 2023) and *C.liuliana* Z. H. Chen, W. Y. Xie & F. Chen (Xie et al. 2023), were reported and a new combination *C.subglabra* (C. Pei) L. X. Ye & B. Y. Ding was proposed (Ye et al. 2018). Thus, to date, a total of 53 *Callicarpa* species have been reported to be distributed in China.

For a long time, we have been puzzled by a species of *Callicarpa* with a quite large population cultivated in Wuhan Botanical Garden, Chinese Academy of Sciences. The species has attracted considerable attention due to features, such as procumbent branches, evergreen leaves, white fruits, scarless nodes and stellate pubescence free fruits. These features make the species distinguishable from any other previously-characterised *Callicarpa* species. In order to further analyse this species, we reviewed the original records and revisited its original source area in March 2023 in Yongshun, Hunan, China. Species specimens were also collected in December 2023 from Hupingshan, Shimen, Hunan, China. Careful literature review combined with comprehensive morphological and molecular characterisation obviously identified this plant as a new species in the genus *Callicarpa* from China.

## ﻿Materials and methods

All available specimens in the genus *Callicarpa* from Hunan Province were retrieved from the China Virtual Herbarium (http://www.cvh.ac.cn/) and JSTOR Global Factory (https://plants.jstor.org/) and examined. Observation and morphological description of the new species was conducted, based on living plants cultivated in Wuhan Botanical Garden and individuals obtained from their type localities. The microstructure of flowers, fruits and glands were observed and photographed using a Nikon SMZ25 stereomicroscope (Nikon, Japan), while additional images were taken with a Canon 90D digital camera (Nikon, Japan). The morphological traits of the new species were described according to previous reports (Fang 1982; Chen and Michael 1994).

Sixteen Chinese *Callicarpa* species (Ma et al. 2023) were selected as representatives for phylogenetic analysis, with *Dasymallateckiana* (F. Muell.) B. J. Conn & Henwood and *Dicrastylisparvifolia* F. Muell. being used as outgroups. Genomic DNA was purified from the fresh plant leaves using the cetyltrimethylammonium bromide (CTAB) method (Doyle and Doyle 1987), then sequenced on an Illunima Novoseq platform in Novogene (https://cn.novogene.com/) to obtain a 5G raw data of paired end reads. The complete nuclear ribosomal sequence and complete chloroplast genome sequence was assembled using Getorganelle1.7.7.0 (Jin et al. 2020) (kmer = 127). Two nuclear DNA markers, ITS and ETS, from the nuclear ribosomal sequence and five chloroplast markers, including *matK*, *rpl32-trnL*, *trnH-psbA*, *psbJ-petA* and *trnS-trnG* intergenic spacer from the chloroplast genome sequence were used to construct the phylogenic relationships of the plant species. The GenBank accession numbers of sequences used in this study are listed in Table 1. All sequences were aligned with MAFFT 7.520 (Rozewicki et al. 2019) and the seven aligned DNA markers were concatenated in Phylosuite 1.2.3 (Zhang et al. 2020). The substitution model was assessed in Phylosuite 1.2.3 (Zhang et al. 2020) using ModelFinder (Kalyaanamoorthy et al. 2017). Bayesian Inference (BI) was carried out using MrBayes (Ronquist et al. 2012) in Phylosuite v.1.2.3 (Zhang et al. 2020). The number of generations run by the Markov chains was set to 2,000,000, with sampling every 1,000 generations and a burn-in of 0.25. Four Markov chains with two runs were performed. The Maximum Likelihood (ML) phylogenetic trees were inferred with IQ-TREE 2.2 (Minh et al. 2020, parameters: -m TEST, -bb 5000 and -bcor 0.90).

**Table 1.** GenBank accession numbers of sequences used in this study.

Taxon	ETS	ITS	*matK*	*psbJ-petA*	*rpl32-trnL*	*trnG(UCC)-trnS(GCU)*	*trnH-psbA*
*C.yongshunensis*	OR271683	OR271683	OR263673	OR263673	OR263673	OR263673	OR263673
*C.luteopunctata*	OM307551	OM333858	OM630179	OM460804	OM501627	OM403853	OM489766
*C.japonica*	ON931539	ON820162	OP032150	OP032157	OP081587	OP032168	OP032163
*C.brevipes*	ON931491	ON820123	OP032115	OM460815	OP081586	OP032173	OP032167
*C.hainanensis*	OM307542	OM333849	OM630173	OM460810	OM501619	OM403845	OM473350
*C.longipes*	OM307549	OM333856	OM630178	OM460805	OM501625	OM403851	OM473340
*C. integerrima var. chinensis*	OM307530	OM333838	OM630183	OM460802	OM501631	OM403857	OM473347
*C.rubella*	OM307557	OM333864	ON964474	OP032159	OM530212	OM403906	OM473339
*C.stoloniformis*	OP032174	OP030615	OP032175	OP032176	OP032177	OP032178	OP032179
*C.poilanei*	OM307603	OP135611	OM530154	OM439786	OM501603	OM403827	OM439784
*C.arborea*	ON931488	ON820119	OP032112	OP032161	OP081584	OP032171	OM489764
*C.candicans*	OM307537	OM333844	OM630169	OM460814	OM501614	OM403840	OM473348
*C.giraldii*	ON931499	ON820130	OP032121	OP032160	OP081585	OP032170	OP032165
*C.nudiflora*	OM307553	OM333860	OM630181	OM460801	OM501629	OM403855	OM489771
*C.longifolia*	ON931514	OM333855	OP032131	OP032162	OP081583	OP032172	OP032166
*C.pentandra*	ON931526	ON820150	OP032141	OP032158	OP081588	OP032169	OP032164
*Dasymallateckiana*	#	#	NC_058334	NC_058334	NC_058334	NC_058334	NC_058334
*Dicrastylisparvifolia*	#	GQ381162	NC_058335	NC_058335	NC_058335	NC_058335	NC_058335

## ﻿Taxonomic treatment

### ﻿ Callicarpa yongshunensis

Taxon classificationPlantaeLamialesLamiaceae

Wen B. Xu, Xiao D. Li & Yan Ling Liu, sp nov.

A49F4F31-1BC0-5BFE-A5E9-7470BC42A8DE

urn:lsid:ipni.org:names:77340494-1

Figs 1 , 2

#### Type.

China. Hunan Province, Yongshun County, Shanmuhe Forestry Farm, growing under the forest by the edge of a valley, 29.16517°N, 109.84132°E, 676 m alt., 16 March 2023(fr.), *W.B. Xu & X.W. Li 230155* (holotype: HIB, barcode no. 0250267, isotype: HIB, barcode no. 0250265).

**Figure 1.**
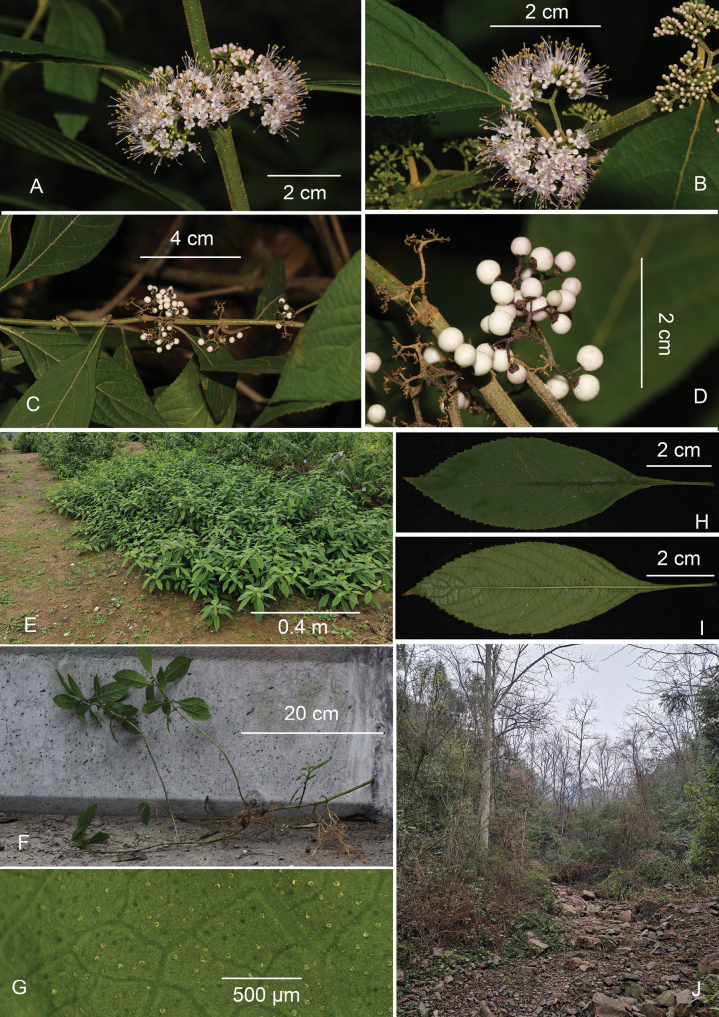
Images of *Callicarpayongshunensis* Wen B. Xu, Xiao D. Li & Yan Ling Liu **A** inflorescence in ventral view **B** inflorescence in lateral view **C** fruit branch in lateral view **D** infructescence in ventral view **E** individual in the fruiting period of wild populations **F** roots developed from the node area of fruit branches **G** glands on the abaxial surface of the leaf **H** leaf, adaxial surface **I** leaf, abaxial surface **J** typical species natural habitat. Photos by Wen-Bin XU and Shu-Hui WANG.

#### Diagnosis.

*C.yongshunensis* is morphologically similar to *C.luteopunctata* H.T. Chang, *C.giraldii* Hesse ex Rehd. and *C.longifolia* Lamk. (Table 2), but differs from *C.luteopunctata*in and *C.giraldii* in being a procumbent shrub (vs. erect shrubs) with evergreen leaf (vs. deciduous leaf) and white fruit (vs. red or purple fruit). Similarly, unlike *C.longifolia*, it has procumbent shrubs (vs. erect shrubs), no transverse scar in the nodes (vs. nodes with a transverse scar) and glabrous mature fruits covered with yellow glands (vs. mature fruit covered with stellate pubescence).

**Table 2.** Morphological comparison of *C.yongshunensis*, *C.luteopunctata*, *C.giraldii* and *C.longifolia* (Fang 1982; Chen and Michael 1994).

Characters	*C.yongshunensis*	*C.luteopunctata*	*C.giraldii*	*C.longifolia*
Life form	procumbent shrub	erect shrub	erect shrub	erect shrub
Leaf habit	evergreen	deciduous	deciduous	evergreen
Leaf size	7.5–16 × 3.5–5.5 cm	7–16 × 2–5 cm	5–17 × 2–10 cm	8–20 × 2–6 cm
nodes with a transverse scar	absent	absent	absent	present
Calyx length	ca. 0.7 mm	ca. 0.7 mm	ca. 1.5 mm	ca. 1 mm
Corolla colour	light red to light purple	purple	purple	pale purple
Corolla length	ca. 1.3 mm	ca. 1.4 mm	ca. 3 mm	ca. 2 mm
Fruit diameter	ca. 2 mm	ca. 1 mm	2.5–4 mm	ca. 1.5 mm
Fruit colour	white	red (Liu et al. 2023)	purple	white
Mature fruit overlay	glabrous with yellow glands	glabrous with yellow glands	glabrescent without glands	stellate pubescent with sparse yellow glands

**Figure 2.**
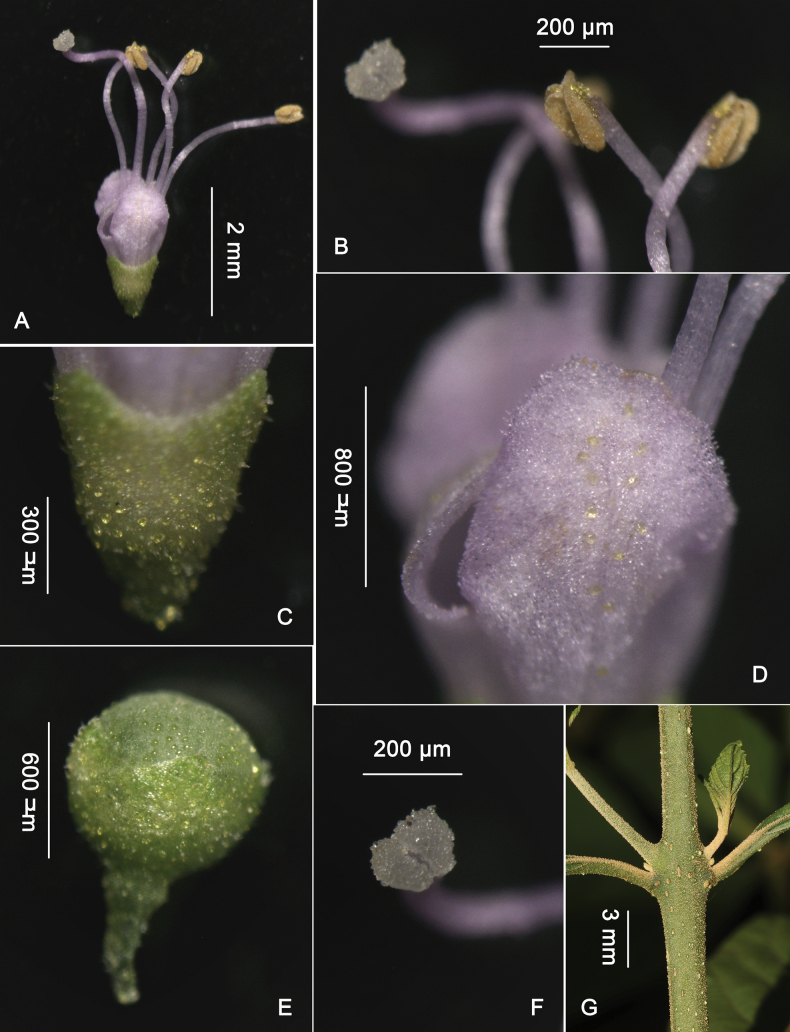
The microstructure of flowers and fruits of *Callicarpayongshunensis***A** flower **B** longitudinally dehiscing anther **C** calyx and attached glands **D** petals and attached glands **E** young fruit and attached glands **F** bifid stigma **G** petiole nodes without transverse scar. Photos by Shu-Hui WANG.

#### Description.

Procumbent shrub without an obvious main stem, up to 1.3 m high, branches slender, often base close to the ground and apex rising, sometimes roots growing from nodes when the nodes reaching the ground, nodes without transverse scar. Branchlets slightly 4-angled, densely covered with yellowish-brown stellate indumentum and yellow glands. Leaf with petiole 1.5–2.5 cm long, blade ovate-elliptic to oblong-elliptic, 7.5–16 × 3.5–5.5 cm, base narrowly cuneate and decurrent, margin serrate, apex acuminate, both surfaces subglabrous (with occasional hairs) and densely covered with small yellowish glands. ***Cymes*** 2–3 cm in diameter, 4–5-bifurcated, densely covered with stellate hairs and yellow glands; peduncle 4–7 mm. ***Calyx*** cup-shaped, 0.6–0.7 mm long, glabrous, sparsely yellow glandular, margin obscurely dentate and apex subtruncate. ***Corolla*** light red to light purple, 1.1–1.4 mm long, petals 4, elliptical, glabrous, slightly covered yellow glands outside. ***Stamens*** strongly exserted, usually twice as long as corolla. ***Style*** nearly as long as stamens, stigma bifid. ***Fruit*** white, 1.7–2.2 mm in diam., glabrous with sparsely yellow glands.

#### Distribution and habitat.

Currently, *C.yongshunensis* has been found in Yongshun and Shimen Counties in Hunan Province, China. It occurs in forests with weak light, at an elevation of 600–680 m. The companion species mainly include *Litseaelongata* (Wall. ex Nees) Benth. et Hook. f., *Camelliacostei* Levl., *Camelliaoleifera* Abel., *Dichroafebrifuga* Lour., *Maesajaponica* (Thunb.) Moritzi. ex Zoll. and *Monachosorumhenryi* Christ.

#### Phenology.

Flowers in cultivated plants at the Wuhan Botanical Garden appeared between May and July, while fruits were observed in both wild and cultivated plants at the Botanical Garden from October to March of the following year.

#### Etymology.

The type specimen of this species was collected from Yongshun County, Hunan Province. Thus, we chose the specific epithet “*yongshunensis*” for this species. Its Chinese name is “永顺紫珠”.

#### Conservation assessment.

The on-going field investigation only identified few populations within narrow altitude ranges. More fieldwork is still warranted to better understand this taxon. According to the guidelines of IUCN Red List Categories and Criteria (IUCN Standards and Petitions Committee 2022), *C.yongshunensis* is assessed as data deficient (DD).

#### Additional specimens examined.

*C.yongshunensis* (Paratypes): China. Hunan Province: Shimen County, Hupingshan National Nature Reserve, Xiahuanglian River, growing by the stream in the valley, 603 m alt., 20 December 2023 (fr.), *Zhenfa Chen 231202* (HIB, barcode nos. 0250264 & 0250266, CSFI, barcode no. 080033). Hubei Province: Cultivated in Wuhan Botanical Garden, Chinese Academy of Sciences, 15 m alt., 20 June 2022 (fl.), W. B. Xu 20230302 (HIB, barcode no. 0233004).

*C.luteopunctata*: China. Sichuan Province: *E. H. Wilson 5100* (IBSC); Emei Mountain, 10 July 1937, *Chong-Shu Qian 6024* (PE); Leshan City, Mount Emei, 7 July 1941, *Wen-Pei Fang 17200* (PE); Leshan City, Mount Emei, shishungou, 4 November 1952, *Ji-Hua Xiong, Xiu-Shi Zhang & Xing-Lin Jiang 33464* (PE); Emei Mountain, near Hongchunping, 1000 m alt., 20 August 1957, *Guang-Hui Yang 56774* (PE); Xuyong County, Shuiwei Town, Guandou Village, evergreen broad-leaved forest, 105°38'16"E, 28°16'39"N, 790 m alt., 8 June 2013, *Xin-Fen Gao, Yun-Dong Gao & Wen-Bin Ju HGX11767* (CDBI); ibid., growing by the stream, under the forest, 105°23'44"E, 28°09'20"N, 14 September 2013, *Wen-Bin Ju & Heng-Ning Deng HGX13448* (CDBI). Yunnan Province: Yongshan County, growing in a ravine, 1400 m alt., 13 July 1932, *H. T. Tsai 51132* (LBG); Jinping County, Mengla, Tuomazhai, 900 m alt., 28 June 2009, *Yunnan expedition team YN-ET40*0 (PE).

*C.giraldii*: China. Hunan Province: Yuanling County, Jiaomuxi, found at the mountain top, 1000 m alt., 23 June 1988, *Wuling team 533* (IBSC); Zhijiang County, muyexi, in ravine, 90 m alt., 3 October 1988, *Wuling team 1773* (IBSC); Xinning County, Ziyun Mountain, 1150 m alt., 20 October 1962, *Lin-Han Liu 15240* (IBSC); Changsha, Lushan, wetland, 400–500 m alt., 2 July 1929, *Shu-Zhi Xin S.H.23* (IBSC); Chengbu County, Dalao Mountain, occurring under dense forest at the mountaintop, 1500 m alt., 12 July 1959, *Pei-Xiang Tan 63685* (IBSC); Sangzhi County, Shadiping, Luojiatai, valley dense forest with a slope of 20°, 1500 m alt., 22, June 1958, *Lin-Han Liu 9056* (PE). Hubei Province: Yingshan County, growing near Miaoergang, subtropical coniferous broad-leaved mixed forest, roadside, 116°02'31.47"E, 30°58'41.70"N, 991 m alt., 10 August 2018, *Xin-Xin Zhu et al. ZXX18584* (KUN). Jiangxi Province: Jiujiang County, mingshan, obtained under the broad-leaved forest in the valley, by the stream, 200 m alt., 3 June 1993, *Ce-Ming Tan 93192* (PE). Guangdong Province: Ruyuan County, Wuzhi Mountain, Shikeng, 750 m alt., 8 August 1983, *Nian-He Xia & Nian Liu 148* (IBSC).

*C.longifolia*; China. Guangdong Province: Zhaoqing, Dinghu Mountain, near Qingyunshi, 22 July 1979, *Hua-Gu Ye 45* (IBSC); Yangshan County, Wuyuan, Yunyong Mountain, in the shade of the valley slopes, 1000 m alt., 6 June 1956, *Liang Deng 1313* (PE). Hainan Province: Sanya, Ganshiling Reservoir, 17 October 1987, *Ze-Xian Li 2611* (IBSC); Wanning County, xinglong, 14 May 1995, *Fu-Wu Xing et al. 6717* (IBSC). Yunnan Province: Xishuangbanna Prefecture, Jinghong City, Mengyang Town, Guanping, by the edge of the forest, 900 m alt., 10 August 1977, *Guo-Da Tao 17611* (HITBC); Cangyuan County, Nanla, Mangka, growing in dense forests within valleys, 1050 m alt., 6 July 1974, *Yan-Hui Li 12620* (HITBC).

##### ﻿Molecular phylogeny

**Figure 3.**
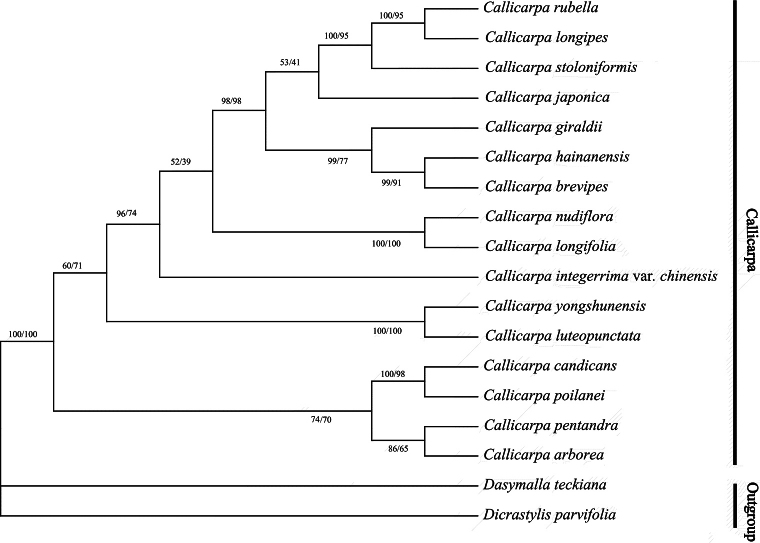
Phylogenetic relationships amongst *Callicarpayongshunensis* and other selected species. The numbers near the nodes are Bayesian posterior probabilities and Maximum Likelihood bootstrap percentages, respectively.

**Figure 4.**
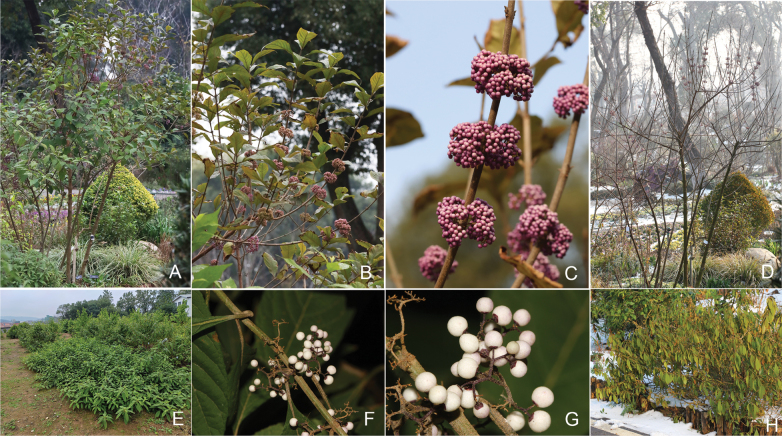
Morphological comparison between *C.luteopunctata* (**A–D**) and *C.yongshunensis* (**E–H**) **A, E** life form showing erect shrub and procumbent shrub **B, F** fruit branch **C, G** fruit colour **D, H** leaf behaviour in winter showing deciduous and evergreen phenotypes (Date: 27 February 2024). Photos by Wen-Bin XU.

Similar topology of the Bayesian and ML trees, both displaying monophyly of the *Callicarpa* was observed (Fig. 3). Overall, three well-supported primary clades were identified with a posterior probability and bootstrap percentages = 1. Notably, *C.yongshunensis* clustered with *C.luteopunctata* and both morphological (Fig. 4) and molecular phylogeny evidence revealed that these two species are closely related. The resulting phylogenetic tree could separate *C.yongshunensis* from *C.giraldii* and *C.longifolia*, while *C.giraldii* clustered with *C.hainanensis* and *C.brevipes*; in addition, *C.longifolia* clustered with *C.nudiflora*. The results further confirmed previous conclusions that the phylogenetic relationship does not always reflect morphological similarity (Chang 1951). For example, *C.brevipes* (section Verticirima), *C.giraldii* (section Eucallicarpa) and *C.hainanensis* (section Verticirima) constituted a subclade with over 0.90 support, while *C.luteopunctata* (section Eucallicarpa) and *C.stoloniformis* (section Verticirima) were tightly clustered with *C.longipes* (section Eucallicarpa) in the phylogenetic tree.

## Supplementary Material

XML Treatment for Callicarpa yongshunensis

## ﻿Additional information

### Author ORCIDs

Wen-Bin Xu https://orcid.org/0009-0002-2645-0053

Xiao-Dong Li https://orcid.org/0009-0002-2910-1449

Shu-Hui Wang https://orcid.org/0000-0003-0067-2344

Ang Liu https://orcid.org/0000-0001-6281-7145

Yan-Ling Liu https://orcid.org/0000-0002-8594-0573

### Data availability

All of the data that support the findings of this study are deposited at NCBI (https://www.ncbi.nlm.nih.gov/).

## References

[B1] Bramley GLC (2011) Distribution patterns in Malesian *Callicarpa* (Lamiaceae). Gardens’ Bulletin (Singapore) 63: 287–298.

[B2] Bramley GLC (2013) The genus *Callicarpa* (Lamiaceae) in the Philippines. Kew Bulletin 68(3): 369–418. 10.1007/s12225-013-9456-y

[B3] Bramley G Bongcheewin B Davies N de Kok RPJ Mabberley DJ Suddee S Walsingham L Wearn JS (2019) Lamiaceae. Flora Malesiana-Series 1. Spermatophyta 23: 1–424.

[B4] Briquet J (1895) Verbenaceae. In: Engler A Prantl K (Eds) Die natürlichen Pflanzenfamilien IV, 3a.Wilhelm Engelmann, Leipzig, 132–182.

[B5] Brown R (1810) Verbenaceae. In: Brown R Esenbeck CGNV (Eds) Prodromus Florae Novae Hollandiae et Insulae Van-Diemen.Richard Taylor & Co., London, 510–514. 10.5962/bhl.title.3678

[B6] Cantino PD Harley RM Wagstaff SJ (1992) Genera of Labiatae: status and classification. In: Harley RM Reynolds T (Eds) Advances in Labiatae science.Royal Botanic Gardens, Kew, 511–522.

[B7] Chang HT (1951) A Review of the Chinese Species of *Callicarpa*. Acta Phytotaxonomica Sinica 1: 269–312.

[B8] Chen SL Michael GG (1994) Verbenaceae. Flora of China Vol. 17. Science Press, Beijing and Missouri Botanical Garden Press, St. Louis, 49 pp.

[B9] Doyle JJ Doyle JL (1987) A rapid DNA isolation procedure for small quantities of fresh leaf tissue. Phytochemical Bulletin 19: 11–15.

[B10] Endlicher S (1836) Genera plantarum secundum ordines naturales disposita, Vol. 1. Apud F. Beck, Vindobonae, 632–639. 10.5962/bhl.title.118772

[B11] Fang WZ (1982) *Callicarpa* L. (Verbenaceae). In: Pei C Chen SL (Eds) Flora Reipublicae Popularis Sinicae.Science Press, Beijing, 24–79.

[B12] Harley RM Atkins S Budantsev AL Cantino PD Conn BJ Grayer R Harley MM de Kok R Krestovskaja T Morales R Paton AJ Ryding O Upson T (2004) Labiatae. In: Kadereit JW (Ed.) Flowering Plants.Dicotyledons. The Families and Genera of Vascular Plants, Vol. 7. Springer, Berlin, Heidelberg, 167–275.

[B13] IUCN Standards and Petitions Committee (2022) Guidelines for using the IUCN Red List Categories and Criteria. Version 15.1. Prepared by the Standards and Petitions Committee. https://www.iucnredlist.org/resources/redlistguidelines/

[B14] Jin JJ Yu WB Yang JB Song Y dePamphilis CW Yi T-S Li D-Z (2020) GetOrganelle: A fast and versatile toolkit for accurate de novo assembly of organelle genomes. Genome Biology 241(21): 241. 10.1186/s13059-020-02154-5 PMC7488116 32912315

[B15] Kalyaanamoorthy S Minh BQ Wong TKF von Haeseler A Jermiin LS (2017) ModelFinder: Fast model selection for accurate phylogenetic estimates. Nature Methods 14(6): 587–589. 10.1038/nmeth.4285 28481363 PMC5453245

[B16] Linnaeus C (1753) Species Plantarum. Impensis Laurentii Salvii: Stockholm, Sweden.

[B17] Liu X Cai HM Wang WQ Lin W Su ZW Ma ZH (2023) Why is the beautyberry so colourful? Evolution, biogeography, and diversification of fruit colours in *Callicarpa* (Lamiaceae). Plant Diversity 45(1): 6–19. 10.1016/j.pld.2022.10.002 36876305 PMC9975479

[B18] Ma ZH Zhang DX (2012) *Callicarpahainanensis*: A new species of *Callicarpa* from Hainan, China. Journal of Systematics and Evolution 50(6): 573–573. 10.1111/j.1759-6831.2012.00229_1.x

[B19] Ma ZH Bramley GLC Zhang DX (2015) Pollen morphology of *Callicarpa* L. (Lamiaceae) from China and its systematic implications. Plant Systematics and Evolution 302(1): 67–88. 10.1007/s00606-015-1244-8

[B20] Ma ZH Su XX Cai HM Su ZW Chen B (2023) *Callicarpastoloniformis* (Lamiaceae), a new species from Southeast China based on morphological characters and phylogenetic evidence. Ecology and Evolution 13(3): e9913. 10.1002/ece3.9913 PMC10034482 36969937

[B21] Minh BQ Schmidt HA Chernomor O Schrempf D Woodhams MD von Haeseler A Lanfear R (2020) IQ-TREE 2: New models and efficient methods for phylogenetic inference in the genomic era. Molecular Biology and Evolution 37(5): 1530–1534. 10.1093/molbev/msaa015 32011700 PMC7182206

[B22] Mint Evolutionary Genomics Consortium (2018) Phylogenomic mining of the mints reveals multiple mechanisms contributing to the evolution of chemical diversity in Lamiaceae. Molecular Plant 11(8): 1084–1096. 10.1016/j.molp.2018.06.002 29920355

[B23] Moldenke HN (1936) A monograph of the genus *Callicarpa* as it occurs in America and in cultivation. Feddes Repertorium Specierum Novarum Regni Vegetabilis 39(10–25): 288–317. 10.1002/fedr.19360391013

[B24] Olmstead RG dePamphilis CW Wolfe AD Young ND Elisons WJ Reeves PA (2001) Disintegration of the Scrophulariaceae. American Journal of Botany 88(2): 348–361. 10.2307/2657024 11222255

[B25] Ronquist F Teslenko M van der Mark P Ayres DL Darling A Hohna S Larget B Liu L Suchard MA Huelsenbeck JP (2012) MrBayes 3.2: Efficient Bayesian phylogenetic inference and model choice across a large model space. Systematic Biology 61(3): 539–542. 10.1093/sysbio/sys029 22357727 PMC3329765

[B26] Rozewicki J Li SL Amada KM Standley DM Katoh K (2019) MAFFT-DASH: Integrated protein sequence and structural alignment. Nucleic Acids Research 47(1): 5–10. 10.1093/nar/gkz342 PMC6602451 31062021

[B27] Schauer JC (1847) Verbenaceae. In: De Candolle A (Ed.) Prodromus systematis naturalis regni vegetabilis, Vol.11. Victoris Masson, Paris, 522–700.

[B28] Wagstaff SJ Olmstead RG (1997) Phylogeny of Labiatae and Verbenaceae Inferred from *rbcL* Sequences. Systematic Botany 22(1): 165–179. 10.2307/2419684

[B29] Wang Q (2019) Flora of Pan-Himalaya Vol. 44 (1). Science Press, Beijing and Cambridge University Press, Cambridge.

[B30] Xie WY Chen F Lin J Mei XD Chen ZH (2023) *Callicarpaliuliana* (Verbenaceae), a New Species from Zhejiang Province. Journal of Hangzhou Normal University 22(3): 262–265. [Natural Science Edition]

[B31] Ye LX Liu LJ Ding BY (2018) Two new combinations of *Callicarpa* (Verbenaceae) from Zhejiang. Journal of Zhejiang Forestry Science and Technology 38(6): 92–94.

[B32] Zhang D Gao FL Jakovlic I Zou H Zhang J Li WX Wang GT (2020) PhyloSuite: An integrated and scalable desktop platform for streamlined molecular sequence data management and evolutionary phylogenetics studies. Molecular Ecology Resources 20(1): 348–355. 10.1111/1755-0998.13096 31599058

[B33] Zhao F Chen YP Salmaki Y Drew BT Wilson TC Scheen AC Celep F Bräuchler C Bendiksby M Wang Q Min DZ Peng H Olmstead RG Li B Xiang CL (2021) An updated tribal classification of Lamiaceae based on plastome phylogenomics. BMC Biology 19(1): 2. 10.1186/s12915-020-00931-z 33419433 PMC7796571

